# Do Parental Hormone Levels Synchronize During the Prenatal and Postpartum Periods? A Systematic Review

**DOI:** 10.1007/s10567-024-00474-7

**Published:** 2024-04-14

**Authors:** Negin Daneshnia, Natalia Chechko, Susanne Nehls

**Affiliations:** 1 Department of Psychiatry, Psychotherapy and Psychosomatics, Medical Faculty, RWTH Aachen, Aachen, Germany; 2 Institute of Neuroscience and Medicine: JARA-Institute Brain Structure Function Relationship (INM-10), Research Center Jülich, Jülich, Germany; 3 Institute of Neuroscience and Medicine, Brain and Behavior (INM-7), Research Center Jülich, Jülich, Germany

**Keywords:** Hormonal synchrony, Coregulation, Linkage, Parental, Transition to parenthood

## Abstract

Physiological synchrony is the phenomenon of linked physiological processes among two or more individuals. Evidence of linkage between dyads has been found among a broad range of physiological indices, including the endocrine systems. During the transition to parenthood, both men and women undergo hormonal changes that facilitate parenting behavior. The present review sought to address the question as to whether hormonal synchronization occurs among expecting or new parents. A systematic literature search yielded 13 eligible records. The evidence of cortisol synchrony during the prenatal period, with additional testosterone, prolactin, and progesterone covariations in the time leading up to childbirth, was found to be most significant. During the postpartum period, parental synchrony was reported for oxytocin, testosterone, and cortisol levels. The implications of these covariations were found to translate into adaptive parenting behaviors and the facilitation of romantic bond. Associations with infant development were also reported, suggesting far-reaching effects of hormonal synchrony outside the parental dyad. The results highlight the importance of physiological interrelatedness during this sensitive period, underscoring the need for further research in this field. In view of the limited data available in this research domain, we have put forward a framework for future studies, recommending the adoption of standardized research protocols and repeated collections of specimens.

For new parents, the transition to parenthood is a transformative period characterized by a host of psychosocial and physiological changes preparing them for their lifelong role. Focusing on maternal alterations, studies have pointed out a variety of neural as well as endocrinological changes that are thought to contribute to motherly attachment and fetal development (Brunton & Russell, 2008; Miranda & Sousa, 2018). Recent research has indicated the role of paternal hormonal changes in adaptive parenting behavior (Storey et al., 2000), underscoring the importance of including both caregivers in studies pertaining to parenting behavior. Fathers play an essential role in the prenatal period, their support, and coregulation with their partner having been found to have effects on maternal mental health, thus potentially shaping infant development (Brumberg & Shah, 2020; Hughes et al., 2020; Saxbe et al., 2018). This coregulation, also known as physiological synchrony or linkage, is the moment-to-moment synchronization of physiological processes among two or more individuals (Butler, 2011). Research has supported the notion of interdependence among romantic dyads, with partners found to mutually influence each other directly and indirectly, and both intentionally and otherwise (Timmons et al., 2015). Thought to have a positive influence on relationship quality, such coregulation has also been found to be associated with greater empathy and overall relationship satisfaction (Hasler & Troxel, 2010; Ruef, 2001). Given the relevance of these changes to parental as well as infant development, the present systematic review sought to investigate whether parental endocrine systems synchronize during the transition to parenthood. We also sought to locate the hormones involved in the process, while pinpointing the temporal dynamics of these covariations. Additionally, we scrutinized possible adaptive or maladaptive effects and their implications for interpersonal and physiological functioning of the dyad.

## Physiological Synchrony Among Romantic Dyads

As there is no universally accepted definition of physiological synchrony, we would like to define the phenomenon as the moment-to-moment synchrony, *linkage, attunement,* or *covariation*, between at least two individuals in their physiological states (Butler, 2011). The Social Baseline Theory proposed by Beckes and Coan (2011) suggests that close relationships are evolutionarily advantageous in that they regulate physiological systems that help maintain bodily homeostasis. Based on this viewpoint, the linkage among romantic partners can be seen as representing an adaptive homeostatic process, in which greater synchrony favors greater stability within the dyad (Timmons et al., 2015). A wide array of studies has found evidence of covariation between pairs among a wide range of physiological indices, including synchronized heart rates, brain activity, or even sleeping time (Timmons et al., 2015). Further, several studies have demonstrated the synchrony of couples’ hypothalamic–pituitary–adrenal (HPA) axis activity, showing synchronous diurnal and cortisol reactivity levels (Liu et al., 2013; Meyer & Sledge, 2020; Saxbe & Repetti, 2010; Timmons et al., 2015), the strength of which being predicated on the length of the relationship (Laws et al., 2015). This notwithstanding, the question as to whether these linkages serve an adaptive or maladaptive purpose remains, given that the findings have been contradictory. While greater synchrony has been reported in dyads who share greater empathy for their partners’ affective states (Ruef, 2001), stronger linkages have been found in pairs prone to more altercations and overall low relationship quality (Liu et al., 2013; Saxbe & Repetti, 2010). Physiological synchrony within romantic relationships thus appears to be a context-specific dyadic process (Timmons et al., 2015), displaying bi-directional effects.

## Endocrinological Changes During the Transition to Parenthood

During the family-formation process, parents display neural and hormonal changes that have been found to be associated with different adaptive parental behaviors (Brunton & Russell, 2008; Diaz-Rojas et al., 2021). Women in particular undergo drastic hormonal shifts during the gestation and postpartum periods, which are vital in the regulation of the physiological processes needed for the maintenance of pregnancy, fetal development, and the facilitation of maternal care (Brunton & Russell, 2008; Kodogo et al., 2019; Miranda & Sousa, 2018). While these changes are predominant and most crucial, research also hints at notable paternal alterations in expecting fathers, which, despite not subserving any physiological functional significance unlike those of the mothers, have been found to promote the aspects of paternal care (Storey et al., 2000). Of all the parenting hormones, the involvement of oxytocin, prolactin, testosterone, and cortisol most frequently accompanies the expression of parenting among both sexes (Feldman & Bakermans-Kranenburg, 2017; Storey & Ziegler, 2016; Wynne-Edwards, 2001). These endocrines undergo intricate fluctuations during the time leading up to childbirth as well as in the postpartum period. While interrelationships are rather complex and multifaceted, they are assumed to reflect the equally complex physiological and behavioral changes associated with parenthood (Storey et al., 2000). Oxytocin, prolactin, testosterone, and cortisol influence and respond to one another in ways that involve complex feedback loops. Testosterone, for instance, interacts with both oxytocin and cortisol, while oxytocin has been found to modulate testosterone release, with social interactions associated with increased oxytocin levels likely impacting testosterone levels (Fragkaki et al., 2017). On the other hand, cortisol, the stress hormone, can influence testosterone production (Brownlee et al., 2005). Chronic stress and elevated cortisol levels may lead to a decrease in testosterone production, affecting mood, energy levels, and reproductive functions (Zitzmann, 2020). Cortisol, in turn, has intricate interactions with oxytocin. While acute stress can temporarily elevate oxytocin levels, chronic stress may disrupt the oxytocin system, leading to decreased sensitivity to the hormone (Neumann et al., 2000; Pierrehumbert et al., 2012).Oxytocin and prolactin often exhibit a positive correlation, particularly during reproductive events such as childbirth (Jonas et al., 2009). While prolactin is found to regulate oxytocin production, both hormones play roles in social bonding, and elevated oxytocin levels may influence prolactin secretion in response to positive social interactions.

While the apparent interrelatedness and complexity of hormonal levels is apparent, studies have also been able to pinpoint specific mechanisms. The neuropeptide oxytocin is associated with indices of social and attachment behaviors (Rajhans et al., 2019), its role in parental behaviors has been noted to be gender-specific, with increased levels in mothers having been found to be linked to nurturing and affection toward the child. Amplifications in fathers, on the other hand, have been observed only during high levels of stimulatory, and not affectionate, contact (Feldman et al., 2010). Besides its particular involvement in labor and lactation, oxytocin has often been linked to motherly attachment and synchrony within the mother–child dyad (Levine et al., 2007). Amplified oxytocin levels in fathers are not as biologically inherent as they are in mothers, although direct interactions with the partner or the infant have been seen to result in notable increases (Gordon et al., 2017). Another neuropeptide involved in this subgroup of parents is prolactin. Aside from its direct involvement in lactation (Svennersten-Sjaunja & Olsson, 2005), maternal prolactin levels increase during pregnancy and through the postpartum period, with steady amplifications during times of direct contact with the infant (Grattan, 2011). Male prolactin levels have been found to be significantly higher in fathers compared to non-fathers (Gettler et al., 2012), with a sustained increase following childbirth, likely fostering father–child attachment (Storey et al., 2000). While neuropeptide changes have been found to be predominant in mothers, research on the steroid hormone testosterone is more well defined with respect to fathers. Compared to non-fathers, paternal testosterone levels are generally lower (Gettler et al., 2011), with further decreases resulting from interactions with the infant and the pregnant partner (Berg & Wynne-Edwards, 2002; Saxbe et al., 2017). Furthermore, increased empathy and sensitivity toward the infant have been seen to be associated with paternal testosterone levels (Fleming et al., 2002; Kuo et al., 2012). Maternal testosterone shifts are less well defined, with reports indicating a general increase during pregnancy, followed by a postpartum decrease (Edelstein et al., 2015; Fleming et al., 1997). Intranasal testosterone administration to nulliparous women has been found to activate brain regions involved in parental care, with increased neural responsiveness to infant cries (Bos et al., 2010), suggesting its involvement in maternal behavior. During pregnancy, maternal cortisol levels naturally increase by two to four times, which is crucial for fetal development (Morsi et al., 2018), and rapidly decrease after labor (Thompson & Trevathan, 2008). It has been found to be associated with subsequent maternal involvement and caregiving behavior (Barrett & Fleming, 2010; Bos, 2016). Research on paternal cortisol changes has demonstrated an increase in the weeks leading up to childbirth, likely reflecting the anticipatory stress related to the event (Storey et al., 2000). Constant cortisol changes have been detected in fathers during the postpartum period, with decreased cortisol levels being linked to more engaging paternal behavior (Storey et al., 2000).

The significant changes seen in the hormone levels of both mothers and fathers during their transition to parenthood lend credence to the notion of linked endocrine systems. In light of the findings pertaining to romantic dyads, a couple’s attunement during pregnancy may be seen as an advantage and an important adaptive process. Given that expecting parents face a critical period of vulnerability, with their new role producing new stressors, conflict areas and responsibilities, synchronized endocrine systems between expecting parents, in line with the social baseline theory, may serve as a protective factor, helping maintain bodily homeostasis during this sensitive period. Seeking to shed light on the phenomenon, the present review explored the roots of these covariations as well as their possible implications for the parental dyad.

## Methods

### Search Strategy

A systematic literature search was performed according to the guidelines of the PRISMA (Preferred Reporting Items for Systematic Reviews and Meta-Analyses) statements (Page et al., 2021). The search was conducted for literature published on the electronic databases of Web of Science (all databases), PubMed and Scopus, using the following keywords: (1) perinatal OR prenatal OR postnatal OR postpartum OR pregnancy AND (2) paternal OR maternal OR couple OR parent OR father OR mother OR partner AND (3) hormone* OR linkage OR synchron* OR testosterone OR estrogen OR prolactin OR oxytocin OR vasopressin OR estradiol OR cortisol.

### Inclusion and Exclusion Criteria

To be considered, studies had to meet the following inclusion criteria: (1) publication between January 1, 1969 and January 30, 2024; (2) peer-reviewed; (3) written in English or German; (4) subjects were couples expecting or having a baby; and (5) the time of measurement was during pregnancy or up until 12 months postpartum. Articles were excluded if they were on nonhumans and if they were case reports, review articles, and meta-analyses.

#### Selected Studies

**Fig. 1:**
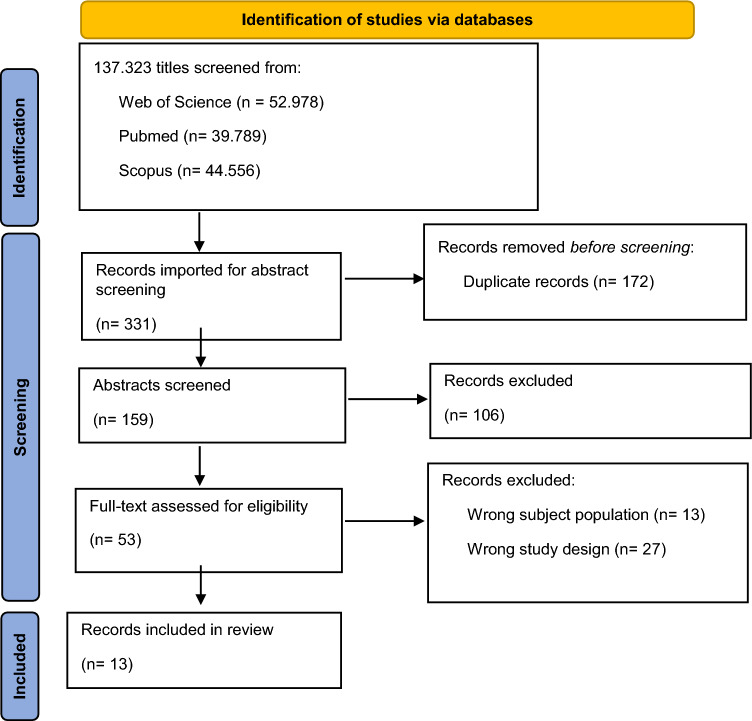
PRISMA flow chart of articles screened

The initial search yielded a total of 137,323 records from the three electronic databases (see Fig. 1 for details). The titles of these articles were screened by a single reviewer (ND), with 331 of them being imported for abstract screening. After the removal of duplicates, 159 records comprised the initial dataset. The screening of the abstracts resulted in 53 studies, likely suitable for inclusion. Discrepancies were discussed and resolved by consensus with a second reviewer (SN), who reviewed a percentage of abstracts. Following the assessment of these full-text records by a single reviewer (ND), 13 were considered eligible for inclusion.

#### Quality Assessment of Included Reviews

The evaluation of study quality was performed using the National Institute of Health Quality Assessment tool aligned with the study setup (National Heart and Blood Institute, 2019). Two reviewers (ND and SN) responded to each of the 14 items in the checklist. The overall quality of the studies was rated as “good,” “fair,” or “poor.” The inter-rater agreement for quality assessment reached 84.62%. Disagreements were resolved through discussion or by consulting a third reviewer. Details regarding the comprehensive ratings per item are presented in Appendix 1.

## Results

### Study Characteristics and Quality

All of the thirteen studies had tested for parental endocrinological linkage in cohabitating partners who were expecting or just had a child. Seven of these studies had been conducted in the USA (Aviv et al., 2023; Cárdenas et al., 2023; Edelstein et al., 2015; Khaled et al., 2021; Saxbe et al., 2015, 2017; Sim et al., 2020), two in Canada (Berg & Wynne-Edwards, 2002; Storey et al., 2000), and two in Israel (Gordon et al., 2010, 2017), with the remaining two having been international studies involving the USA, the United Kingdom, and the Netherlands (Braren et al., 2020, 2021). The majority of studies involved a prospective longitudinal design, while four conducted a cohort study (Aviv et al., 2023; Braren et al., 2020, 2021; Storey et al., 2000). The quality of the included studies was primarily good; ten studies were deemed of good quality, one was deemed to be of fair quality, while the remaining two were deemed of poor quality (National Heart and Blood Institute, 2019; full quality assessment available in Appendix 1).

#### Sample Characteristics

The sample sizes ranged from 9 to 385 couples, resulting in an overall sample pool of 2301 subjects. For both males and females, the total age range was 20–42. The majority of the samples (84.61%) were expecting their first child, were married or engaged, Caucasian, and had earned at least a college degree. For a detailed overview of the sample characteristics, please see Table 1.

**Table 1:** Sample characteristics, sorted alphabetically

References	Country	Subjects (*N* = couples)	Age females (mean or median)	Age males (mean median or range)	Number of children
Aviv et al. (2023)	USA	91	NR	33.35 (= mean)	1
Berg & Wynne-Edwards (2002)	Canada	9	31 (= median)	33 (= median)	1
Braren et al. (2020)	USA, UK and the Netherlands	385	32.42 (= mean)	33.90 (= mean)	1
Braren et al. (2021)	USA, UK and the Netherlands	358	32.36 (= mean)	34.02 (= mean)	1
Cárdenas et al. (2023)	USA	98 (prenatal), 78 (postpartum)	31.22 (= mean)	33.21 (= mean)	1
Edelstein et al. (2015)	USA	29	29.41 (= mean)	30.48 (= mean)	1
Gordon et al. (2010)	Israel	80	27.24 (= mean)	29.45 (= mean)	1
Gordon et al. (2017)	Israel	80	27.72 (= mean)	29.28 (= mean)	1
Khaled et al. (2021)	USA	82	31.3 (= mean)	33.3 (= mean)	1
Saxbe et al. (2015)	USA	122	29.31 (= mean)	31.75 (= mean)	1 to 3
Saxbe et al. (2017)	USA	27	29.19 (= mean)	30.33 (= mean)	1
Sim et al. (2020)	USA	29	29.07 (= mean)	30.1 (= mean)	1
Storey et al. (2000)	Canada	31 4 groups (early prenatal *n* = 12; late prenatal *n* = 8; early postnatal *n* = 9; late postnatal *n* = 8)	25–40 (= range)	25–40 (= range)	1 or more

### Hormone Assessments

The studies measured endocrinological linkage by utilizing either blood plasma (Aviv et al., 2023; Gordon et al., 2010, 2017; Storey et al., 2000) or saliva samples (Berg & Wynne-Edwards, 2002; Braren et al., 2020, 2021; Cárdenas et al., 2023; Edelstein et al., 2015; Khaled et al., 2021; Saxbe et al., 2015, 2017; Sim et al., 2020). Six studies assessed merely cortisol (Braren et al., 2020, 2021; Khaled et al., 2021; Saxbe et al., 2015; Storey et al., 2000), three studied testosterone (Cárdenas et al., 2023; Saxbe et al., 2017; Sim et al., 2020), one prolactin (Aviv et al., 2023), and another one oxytocin (Gordon et al., 2010). The remaining records focused on several hormones simultaneously, including testosterone, cortisol, estradiol, prolactin, progesterone, and oxytocin (Berg & Wynne-Edwards, 2002; Edelstein et al., 2015; Gordon et al., 2017). Most of the studies sought to control for possible hormonal fluctuations by either incorporating a standardized time of measurement (Aviv et al., 2023; Cárdenas et al., 2023; Gordon et al., 2010, 2017; Khaled et al., 2021; Storey et al., 2000) or by measuring at preassigned time points over the day (Braren et al., 2020, 2021; Edelstein et al., 2015; Saxbe et al., 2015, 2017; Sim et al., 2020). Only one study did not integrate any measures to control for possible fluctuations (Berg & Wynne-Edwards, 2002). The measurement time points varied greatly across the studies, although most focused on the prenatal period.

### Hormonal Linkage

The present systematic search resulted in the acquisition of evidence for hormonal synchronization between cohabitating parents during the prenatal and postnatal periods. For a detailed overview of the findings, please see Table 2*.*

**Table 2:** Summary of findings, sorted from prenatal to postpartum periods

References	Design	Time of measurement	Hormone(s) assessed	Specimen	Standardized sampling time	Statistical measure used	Relevant findings	Additional outcome variable	Linkage prenatal or postpartum
Aviv et al. (2023)	Cohort study 1 sample at 1 time	~ 20–35-weeks of gestation	Prolactin	Plasma	Late afternoon	Pearson correlation coefficient and Multilevel modeling	Positive prenatal correlation for prolactin (*r* = 0.225, *p* = .039). This also held in a regression model (*b* = 0.05, *ß* = 0.05, *p* = .05)	No	Prenatal
Berg & Wynne-Edwards (2002)	Longitudinal study 229 time- and date-matched samples from 7 time points	From second trimester up to 2–3 months postpartum (second trimester, third trimester, last month, last week, 1 week postpartum, 1 month postpartum, 2–3 months postpartum)	Testosterone, cortisol, estradiol	Saliva	No	Pearson correlation coefficient	Positive prenatal correlation for cortisol (*r* = 0.24 ± 0.10, *p* = .04). No other significant findings	No	Prenatal
Braren et al. (2020)	Cohort study 3 samples on 2 consecutive days	~ 36 weeks of gestation	Cortisol	Saliva	Sample 1: immediately upon waking, sample 2: 30 min. after first sample, sample 3: immediately before bedtime	Multilevel modeling	Significant small positive within-dyad linkage between maternal and paternal cortisol (*b* = 0.04, *ß* = 0.07, *p* = .04)	Cortisol linkage was significantly associated with maternal psychological stress (*b* = 0.13, ß = 0.18, *p* < .001), not paternal stress	Prenatal
Braren et al. (2021)	Cohort study 3 samples on 2 consecutive days	~ 36 weeks of gestation	Cortisol	Saliva	Sample 1: immediately upon waking, sample 2: 30 min. after first sample, sample 3: immediately before bedtime	Multilevel modeling	Significant positive within-dyad linkage between maternal and paternal cortisol (*b* = .012, *ß* = .02, *p* < .001)	Cortisol linkage positively correlated with paternal sensitivity at 14 months (*p* = .11). High cortisol linkage predicted infant executive function performance at 24 months in the presence of high paternal cortisol (*ß* = 0.29, 95% CI [0.11, 0.46]	Prenatal
Edelstein et al. (2015)	Longitudinal study 2 samples from 4 time points	~ 8-week intervals from weeks 12, 20, 28, and 36 of gestation	Testosterone, cortisol, estradiol, and progesterone	Saliva	12:30 to 18:30; 20-min. interval between the 2 samples per time point	Pearson correlation	Significant averaged within-dyad linkage across all time points for progesterone (*p* < .01), cortisol (*p* < .05), and testosterone (*p* < .10). No significant findings for estradiol	No	Prenatal
Khaled et al. (2021)	Longitudinal study 6 samples from 1 time point	Prenatal sample collection during 20–25 weeks of gestation, consisting of 6 samples: at baseline; baseline + 20 min.; baseline + 35 min.; baseline + 50 min.; baseline + 70 min.; baseline + 90 min	Cortisol	Saliva	At or after 14:00	Multilevel Modeling	Positive within-dyad associations for cortisol linkage (mothers, *ß* = 0.17(*p* < .01); fathers, *ß* = 0.22, (*p* < .01)	Stronger cortisol linkage in couples was significantly associated with less negative conflict behavior for mothers (*ß* = -0,10, *p* = .00) and fathers (*ß* = -0,10, *p* = .02) on the day of prenatal sample collection. Stronger prenatal cortisol linkage was associated with fewer paternal PPD symptoms (*ß* = −0,24, *p* = .03). No significant association with maternal PPD	Prenatal
Saxbe et al. (2017)	Longitudinal study 2 samples from 4 time points	~ 8-week intervals from weeks 12, 20, 28, and 36 of gestation	Testosterone	Saliva	12:30 to 18:30; 20-min. interval between the 2 samples per time point	Pearson Correlation and Multilevel Modeling	Significant within-dyad associations in weeks 28 and 36 of gestation (*p* < .05); no significant associations for weeks 12 and 20	Paternal coregulation with partner during the four time points was predictive of his postpartum relationship investment (*p* < .05). No such association was found for maternal investment	Prenatal
Sim et al. (2020)	Longitudinal study 2 samples from 4 time points	~ 8-week intervals from weeks 12, 20, 28, and 36 of gestation	Testosterone	Saliva	12:30–18:30; 20-min. interval between the 2 samples per time point	Pearson correlation	Significant positive within-dyad association at 36 weeks of gestation (*r* = 0.44*, p* = .03); no significant correlations for the remaining time points	No	Prenatal
Storey et al. (2000)	Cohort study 1 sample at 1 of 4 times	Sample divided into 4 groups: 1 = 16–35 weeks of gestation; 2 = 37–40 weeks of gestation; 3 = up to 3 weeks postpartum; 4 = 4–7 weeks postpartum	Cortisol	Plasma	16:00–20:00	Pearson correlation	Significant positive within-dyad association for cortisol in the combined (group 1 and 2) prenatal groups (*r* = 0.44, *p* = .01). No other significant combined associations	No	Prenatal
Gordon et al. (2010)	Longitudinal study 2 samples from 2 time points	First postpartum weeks and 6 months postpartum	Oxytocin	Plasma	16:00–20:00	Pearson correlation	Significant within-dyad linkage in the first weeks (*p* < .05) and 6 months postpartum (*p* < .01)	No	Postpartum
Gordon et al. (2017)	Longitudinal study 2 samples from 2 time points	1 and 6 months postpartum	Testosterone and oxytocin	Plasma	16:00–20:00	Pearson correlation	Significant within-dyad correlations in oxytocin levels for both time points (t1: *r* = 0.30, *p* = .011; t2: *r* = 0.28, *p* = .045). No significant associations for testosterone	No	Postpartum
Saxbe et al. (2015)	Longitudinal study 1390 samples collected over 242 sampling days	During first 2 years postpartum (female subjects, who got pregnant during the study duration, were assessed during their second or third trimester, accounting for 44 matched days in total)	Cortisol	Saliva	Sample 1: immediately upon waking, sample 2: 30 min. after first sample, sample 3: immediately before bedtime	Multilevel Modeling	Significant within-dyad linkage for cortisol (mothers, *ß* = 0.46, *t* = 9.35, *p* = .01; fathers, *ß* = 0.54, *t* = 12.07, *p* = .001)	Couples with higher relationship aggression at 1-year postpartum had stronger cortisol linkage (*p* < .05)	Postpartum
Cárdenas et al.(2023)	Longitudinal study 6 samples from 2 time points	~ 28-weeks of gestation and ~ 7 months postpartum; Both sample collection times consisted of 6 samples: at baseline; baseline + 20 min.; baseline + 35 min.; baseline + 50 min.; baseline + 70 min.; baseline + 90 min.; here, the first, third, and sixth sample were used for analysis	Testosterone	Saliva	14:00–18:00	Bayesian multilevel modeling	Positive within-dyad associations for testosterone linkage during prenatal (γ01 = 0.69, 95% CI [0.19, 1.20]) and postpartum period ( γ01 = 1.33, 95% CI [0.71, 1.94])	Stronger prenatal couple linkage predicted higher ratings of paternal postpartum relationship quality (γ04 = 0.03, 95% CI [0, 0.07])	Prenatal and postpartum

#### Cortisol

Most of the evidence was found with respect to cortisol linkage, as all of the seven studies reported significant positive associations within the couples. Consistent synchronizations between maternal and paternal cortisol levels had been found in the prenatal period (Berg & Wynne-Edwards, 2002; Braren et al., 2020, 2021; Edelstein et al., 2015; Khaled et al., 2021; Storey et al., 2000) and also in the postpartum period (Saxbe et al., 2015). While most studies merely reported a significant positive within-couple association, some studies established links to a number of other variables, showing stronger cortisol linkage among couples with higher maternal stress (Braren et al., 2020) and among partners with greater aggression in postpartum relationship (Saxbe et al., 2015). Contradicting the latter observation, Khaled et al. (2021) found stronger interrelated cortisol levels to be linked to fewer negative conflict behaviors in both mother and father, while simultaneously predicting less paternal postpartum depression symptoms. Prenatal cortisol linkage was additionally found to be associated with paternal sensitivity at 14 months postpartum and infant executive functions at 24 months, the latter being moderated by the average paternal, and not maternal, cortisol levels (Braren et al., 2021).

#### Testosterone

The covariation in couples during their transition to parenthood was assessed also in terms of their testosterone levels. Out of the six studies, two did not report any significant association either during the prenatal or during the postpartum period (Berg & Wynne-Edwards, 2002; Gordon et al., 2017). In three studies, the interrelatedness of testosterone levels had been assessed prenatally at an eight-week interval between the first and last trimesters (Edelstein et al., 2015; Saxbe et al., 2017; Sim et al., 2020). Here, the couples had displayed averaged prenatal levels that correlated significantly (Edelstein et al., 2015), as well as at 28 (Saxbe et al., 2017) and 36 weeks of gestation (Saxbe et al., 2017; Sim et al., 2020), thus showing signs of coregulation toward the end of pregnancy. Paternal prenatal hormonal synchronization with their partners was further predictive of their relationship investment during the postpartum period, which, however, was not the case for maternal synchronicity (Sim et al., 2020). Additionally, one study assessed parental testosterone synchronizations in the prenatal and postpartum period, namely at 28 weeks of gestation and seven months after childbirth (Cárdenas et al., 2023). Between-couple levels were found to be significantly linked for both time points, with prenatal synchrony being predictive of paternal postpartum relationship quality.

#### Oxytocin

Two studies investigated the interrelatedness of couples’ oxytocin levels during their postpartum period (Gordon et al., 2010, 2017), finding the relevant levels to be significantly linked at both time points, namely in the first weeks of postpartum and twenty-four weeks after childbirth.

#### Estradiol, Progesterone, and Prolactin

Two studies investigated the coregulation of estradiol among couples during the prenatal period (Berg & Wynne-Edwards, 2002; Edelstein et al., 2015) and found no significant associations. Only one study assessed the prenatal progesterone linkage (Edelstein et al., 2015) by examining the expectant couples longitudinally at four time points during their prenatal period and found significantly positive within-couple associations. The couple covariation of prolactin was assessed by one study, finding evidence for its synchrony during the prenatal period (Aviv et al., 2023).

## Discussion

The sensitive period of pregnancy represents a transformative time in a couple’s relationship timeline, accompanied by myriad hormonal changes in both woman and man. While alterations are inherently more predominant and vital in mothers, expectant fathers also display hormonal shifts. Instead of adopting the traditional mother–child dyad approach, considering families as multifaceted systems, within which individuals have reciprocal effects on one another, may facilitate the understanding of probable mutual interrelations.

The literature search for studies on synchronized hormonal levels among couples during the prenatal and postpartum periods identified thirteen studies with relevant evidence, with the majority of findings hinting at covarying parental hormonal levels in both the prenatal and postpartum periods. The evidence of linked prenatal endocrine systems was reported for cortisol (Berg & Wynne-Edwards, 2002; Braren et al., 2020, 2021; Edelstein et al., 2015; Khaled et al., 2021; Storey et al., 2000), testosterone (Cárdenas et al., 2023; Edelstein et al., 2015; Saxbe et al., 2017; Sim et al., 2020), prolactin (Aviv et al., 2023), and progesterone (Edelstein et al., 2015), with significant postpartum linkage in oxytocin (Gordon et al., 2010, 2017), testosterone (Cárdenas et al., 2023), and cortisol levels (Saxbe et al., 2015). Furthermore, five of the thirteen studies reported on significant secondary outcome variables inside the dyad itself (Braren et al., 2020; Cárdenas et al., 2023; Khaled et al., 2021; Saxbe et al., 2015, 2017), with one addressing relevant effects on infant development (Braren et al., 2021). More specifically, higher hormonal linkage within the parental dyad has been associated with higher relationship aggression (Saxbe et al., 2015), paternal postpartum relationship outcomes (Cardenás et al., 2023; Saxbe et al., 2017), fewer paternal postpartum depressive symptoms and increased paternal sensitivity (Khaled et al., 2021), as well as levels of perceived stress in mothers (Braren et al., 2020). Interestingly, associations outside the dyad suggest the importance of parental hormonal synchrony in the long run, as higher synchrony was found to be predictive of infant cognitive development two years postpartum (Braren et al., 2021).

### Parental Hormonal Synchrony in the Prenatal Period

In line with previous research on synchronized endocrine systems among romantic dyads (Timmons et al., 2015), the present review found the most significant evidence of parental linkage in the prenatal basal cortisol levels. Here, covariations were reported as soon as 12 weeks and up to 36 weeks of gestation. Studies have repeatedly reported linked cortisol activity and reactivity in romantic dyads (Meyer & Sledge, 2020; Timmons et al., 2015), with more profound synchrony among distressed pairs (Levenson & Gottman, 1983; Liu et al., 2013). These associations likely reflect the increased psychosocial stress load toward the end of pregnancy as the couple prepares for the baby to arrive. Conceptually, the magnitude of linkage goes far beyond the reflection of stress experiences, as cortisol synchrony may display a couple’s adaptive ability to coregulate each other’s physiological stress response (Timmons et al., 2015). Better coregulation buffers against the negative strains experienced by expecting parents, thus corroborating the widely held notion of its adaptive purpose in homeostatic regulation. Besides its key role in stress responses, cortisol has also been frequently associated with adaptive parental behavior such as greater involvement and caregiving (Barrett & Fleming, 2010; P. Bos, 2016), while greater synchrony is linked to the aspects of physical proximity and time spent together (Laws et al., 2015; Saxbe & Repetti, 2010). Covariation among expecting parents may therefore suggest a growing affinity toward one another during their transition, while mutually promoting the adaptation to their forthcoming role. Given the range of effects cortisol linkage is likely to have on expecting parents, it may be safe to assume that, rather than suggesting any straightforward association, these effects are multi-directional (Timmons et al., 2015).

The evidence of prenatal testosterone interrelatedness, on the other hand, was ambiguous. Here, two studies did not report significant prenatal within-couple associations (Berg & Wynne-Edwards, 2002; Gordon et al., 2017). Despite the low number of subjects and infrequent specimen collection, which could account for the lack of evidence, there was partial support for parental covariation (Cárdenas et al., 2023; Edelstein et al., 2015; Saxbe et al., 2017; Sim et al., 2020). Interestingly, prenatal testosterone linkage was frequently reported during the last trimester (Cárdenas et al., 2023; Saxbe et al., 2017; Sim et al., 2020), suggesting greater coregulation toward the end of pregnancy. Moreover, there was evidence of interrelated bi-directional effects of cortisol and testosterone (Mehta & Josephs, 2007), explaining the observed linkage. From an evolutionary perspective, these findings may indicate an adaptive parenting process, particularly with respect to the father. Generally lower paternal testosterone levels, compared to non-fathers, are thought to be related to parenting behavior, such as increased empathy, sensitivity, and affection toward the infant (Fleming et al., 2002). Most importantly, however, these alterations indicate a shift from a mating behavior to a paternal one. This is supported by the observations of paternal covariation before childbirth to be predictive of the fathers’ subsequent relationship investment (Saxbe et al., 2017) and self-reported relationship quality (Cárdenas et al., 2023), with no associations found with respect to the mothers. Pregnancy itself marks a sensitive period for mothers, during which they are more attuned to affective and stress-related cues from others (Raz, 2014; Senese et al., 2018). Thus, paternal covariations are likely to be adaptive not only in terms of promoting more sensitivity upon the baby’s arrival, but also with respect to the fathers’ attitude toward their partners in the time around childbirth.

The records on prenatal estradiol linkage did not indicate synchronized parental endocrine systems (Berg & Wynne-Edwards, 2002; Edelstein et al., 2015). Again, this could be due to the small sample pool and unstandardized study protocols. Additionally, maternal estradiol levels were found to increase markedly during pregnancy, with the highest concentrations observed just before labor (Fleming et al., 1997; Storey et al., 2000). These shifts are crucial for the physiological changes necessary for a healthy pregnancy and childbirth (Albrecht & Pepe, 2009). Paternal estradiol levels, on the other hand, displayed only subtle shifts, with reported declines in the period leading up to childbirth (Edelstein et al., 2015). These inherent hormonal fluctuations may explain the lack of evidence with respect to prenatal estradiol synchrony. One study examined prolactin covariation among expecting parents (Aviv et al., 2023), finding support for couple synchrony approximately at 28 weeks of gestation. This again suggests shared endocrine levels in the last trimester and just upon the baby’s arrival. While the evidence on prolactin synchrony in the prenatal period is scarce, it does suggest paternal adaptions to parenthood. That is, as maternal prolactin levels significantly increase during pregnancy (Grattan, 2011), whereas paternal levels remain relatively constant throughout, displaying comparatively smaller increases just before childbirth (Storey et al., 2000). Given the substantiated association between heightened prolactin levels and paternal caregiving (Fleming et al., 2002), synchrony toward the end of pregnancy might mirror the process of paternal preparation for fatherhood. However, more research is needed to substantiate that link.

The evidence of prenatal covariation for other parental hormones was also scarce, with only two records on progesterone linkage during the prenatal period (Berg & Wynne-Edwards, 2002; Edelstein et al., 2015). Despite established links to maternal behavior (Glynn et al., 2016), the evidence of progesterone’s impact on paternal parenting can only be inferred from cross-species studies involving male mice, where low progesterone has been found to reduce aggression toward the infant (Schneider et al., 2003). Its role in social bonding, however, has been more evident, with feelings of emotional intimacy being found to amplify progesterone levels, with additional increases in one’s desire to bond and to help others (University of Michigan, 2009). Parental progesterone synchrony may therefore reflect greater intimacy and attachment while, at the same time, facilitating paternal sensitivity.

The evidence of linked prenatal endocrine systems supports the notion of these synchronizations being conducive to the adaptation to parenthood, while promoting the romantic bond. In evolutionary terms, natural selection favors pairs that engage in coordinated parenting (Andersson & Iwasa, 1996). Choosing a hormonally compatible mating partner is thus likely to benefit the reproductive process and its success. More precisely, the hormonal covariation may foster the pair bond, which in turn may promote the formation and maintenance of pair-bonding behaviors, and subsequently the pair’s survival and reproductive success. Furthermore, the mechanism of prenatal endocrine linkage itself is conceptualized on the basis of the inherent nature of maternal hormonal changes during pregnancy, serving both the mother and the fetus (Kodogo et al., 2019; Miranda & Sousa, 2018). Paternal changes, however, are not a biological default, playing, instead, a supportive role by helping fathers adapt to parenting behaviors. Therefore, the phenomenon of prenatal synchronization among couples is likely a matter of the extent of paternal linkage to the maternal hormone levels, and not covariation within the pair per se.

### Parental Hormonal Synchrony in the Postpartum Period

Several studies investigated the new parents’ endocrine systems and the possible linkage thereof following childbirth. Here, evidence of synchrony was found for oxytocin levels up to six months after childbirth (Gordon et al., 2010, 2017). A large body of research supports the critical role of oxytocin in the formation of the parent–child bond (Gordon et al., 2017; Levine et al., 2007), with observed amplifications upon direct contact with the infant (Feldman et al., 2010; Levine et al., 2007). Within-couple oxytocin linkage during the postpartum period likely supports the emergence of parental bonding and attunement to the infant during the family-formation phase. Moreover, this process may even promote attachment in the romantic dyad outside of the parent–child framework. Coregulated oxytocin levels after childbirth may promote feelings of closeness, connection and empathy during this period of transition, facilitating greater stability within the dyad. In line with that, cross-species studies on prairie vole pairs have found stronger oxytocin synchrony to be associated with the strength of the pair bond (Ross & Young, 2009), supporting the notion of its evolutionarily advantageous purpose. Barring partial support for testosterone linkage before childbirth, findings during the postpartum period were ambiguous. Here, one study reported significant parental interrelatedness at approximately seven months postpartum (Cárdenas et al., 2023), while another found no support (Gordon et al., 2017). As opposed to the established postpartum paternal declines (Gettler et al., 2011; Gray et al., 2006), relatively little is known about maternal testosterone changes, impeding the query of synchrony. While future research should be aimed at exploring the matter, findings from cross-species studies indicate that within-couple testosterone synchrony may dynamically vary depending on the situation (Hirschenhauser, 2012). More specifically, a higher prenatal linkage has been found to relate to the reproductive success in geese, whereas lower synchrony is associated with longer bond duration. Within-pair testosterone covariation may thus have a time- and context-specific purpose, similarly to cortisol. Saxbe et al. (2015) found evidence of postpartum cortisol linkage two years after childbirth. In line with the synthesized findings of the prenatal period, a pair’s postpartum cortisol linkage may reflect their ability to coregulate each other’s physiological stress response (Timmons et al., 2015), while promoting adaptive parenting behaviors (Barrett & Fleming, 2010; Bos, 2016). Given the continuous substantiation of cortisol interrelatedness, future research should examine its role in the postpartum period more extensively.

### Postpartum Outcomes of Parental Hormone Synchrony

While most records aimed at merely clarifying the issue of synchronized parental endocrine systems, others examined the dyadic associations in a couple’s attunement and postpartum outcomes. As regards cortisol covariation, it was identified as a moderator for maternal stress (Braren et al., 2020) with the magnitude of linkage being found to be moderated by postpartum relationship aggression (Saxbe et al., 2015). On the other hand, cortisol synchrony was found to serve as a protective factor by being associated with lower levels of conflict-inducing behaviors in both partners (Khaled et al., 2021). These contradicting findings are very much in line with previous work (Liu et al., 2013; Saxbe & Repetti, 2010) and underline the conjecture that the implications of cortisol linkage within romantic dyads are context-dependent (Timmons et al., 2015). Moreover, the associations of cortisol itself are multifaceted and anything but straightforward. While some studies propose a positive relationship, where heightened cortisol levels are directly linked to heightened aggression and the physiological response to challenging situations (Feinberg et al., 2011; van Bokhoven et al., 2005), others indicate that lower cortisol levels may be linked to increased hostility (Shoal et al., 2003). These bi-directional effects preclude the possibility of drawing definitive conclusions in this context, and determining universally applicable connections, indicating, instead, a basis for the contradictory findings.

Additionally, greater testosterone couple covariation was identified as a predictor of greater paternal, but not maternal, postpartum relationship quality and investment (Cárdenas et al., 2023; Saxbe et al., 2017). While further investigations are needed to explore the concrete mechanisms behind these effects, that appear to be linked to fathers’ postpartum relationship outcomes only, one possible explanation lies in the paternal endocrinological adaptions as a preparation to parenthood itself. Lower testosterone levels, as compared to non-fathers, have long been hypothesized to mirror the transition from mating to parenting behaviors (Gettler et al., 2011). Moreover, lower male testosterone levels have been suggested to promote nurturing behavior (Wingfield et al., 1990) and greater investment in family relationships (Kuo et al., 2018), supporting the sex specific postpartum outcome findings.

Interestingly, the effects go far beyond the mere moment-to-moment synchronization, given that higher cortisol linkage during the prenatal period is predictive of less paternal depression symptoms six months after childbirth (Khaled et al., 2021). The family-formation process itself marks a critical period of vulnerability, when many experience their first major depressive episode (Stowe & Nemeroff, 1995), which is referred to as postpartum depression (PPD). A large body of research proposes the dysfunction of the HPA axis to be a potential biomarker for PPD (Jolley et al., 2007; Stickel et al., 2021). Cortisol covariation during the postpartum period may help buffer against paternal vulnerability to affective disorders. Moreover, higher cortisol synchrony has been found to be significantly associated with paternal sensitivity 14 months postpartum, in addition to being predictive of infant executive functioning at 24 months postpartum (Braren et al., 2021), the latter being moderated by the presence of high paternal, and not maternal, cortisol levels. This intriguing observation highlights the magnitude of the paternal impact on the whole family system. The far-reaching effects of cortisol synchrony support the notion that it serves an adaptive and protective purpose, with paternal synchronization to the maternal endocrine system appearing to have a significant mediating role. This finding dovetails with the proposed bio-behavioral synchrony model by Feldman (Feldman, 2012), which views parental synchrony as a template and necessity for the infant. More specifically, linkage is viewed as an intergenerational process, in which parental synchrony has far-reaching implications for infants’ psychosocial and physiological development. This cross-generational transmission supports the assumed evolutionary advantage of physiological synchrony.

### Limitations and Future Directions

The studies reviewed here had several limitations, which need to be pointed out. First, the samples consisted of predominantly white dyads with middle to high socioeconomic status, constraining the generalizability of the findings to a narrow demographic. More research is needed to scrutinize how physiological synchrony affects parents in other populations. Moreover, the inherently fluctuating nature of the endocrine levels acts as a barrier to the categorical question of their synchrony. The multifactorial impact on hormonal variations precludes the possibility to control for all the relevant factors. While only one study did not incorporate a standardized time for measurement (Berg & Wynne-Edwards, 2002), the time and number of specimen collection varied greatly across the studies, thus might not fully reflect the hormonal changes and the linkage thereof. Despite an overall well-sized sample pool, some studies included a low number of participants, again impeding the findings’ generalizability. Future research investigating parental endocrine linkage should incorporate standardized study protocols aimed at larger sample pools to prevent the limitation of statistical power. While the reported within-couple covariations display a link between maternal and paternal hormone levels, neither any directionality nor causality can be inferred from the data. Future cross-sectional designs, incorporating more advanced statistical approaches to examine potential moderator effects, may help disentangle the complex process of hormonal synchrony. In this context, multilevel modeling may prove more advantageous compared to the correlation coefficients used in most of the reviewed studies.

### Establishing a Gold Standard for Hormonal Synchronizations

While there may not be a single universally accepted gold standard for assessing longitudinal hormonal fluctuations and synchronizations, there are best practices that researchers commonly follow to ensure the reliability and validity of their findings. Based on these, we propose the following framework for future data collection on parental endocrinological synchrony. Building on previous hormonal research, we incorporate new suggestions with a focus on validation, control for fluctuations and analytic precision. We believe that establishing of a standard framework can ensure the scientific rigor required to enhance our understanding of the complexity of hormonal synchronizations in a longitudinal setting.

1. *Consistent and standardized protocols* To establish a robust foundation, future studies should implement consistent and standardized protocols for sample collection and storage. We suggest the prior establishment of a study protocol, where researchers specify the concrete approach to the respective research question. Inclusion and exclusion criteria should be established beforehand, while the time sequence of specimen collection should be defined a priori. The adherence to a prespecified study protocol ensures the generalizability, validity, and replication of findings.
2. *Specimen Collection* The complex dynamics of endocrinological fluctuations impede the study thereof. While serum testing is a common approach to determine hormonal levels, it is usually not used for multiple testing, given its invasive nature. We suggest the utilization of an established method such as the analysis of salivary hormones, which has proved to be accurate in determining the values of estradiol, progesterone and cortisol (Kells & Dollbaum, 2009), as well as testosterone (Wang et al., 2019). Easy to assess and non-invasive, saliva assays capture moment-to-moment fluctuations (Dabbs, 1990; Dettenborn et al., 2012; Shirtcliff et al., 2015), enabling repeated measurements that not only obtain a reliable baseline measure, but also, unlike blood samples, obtain moment-to-moment corrections.
3. *Saliva Sampling Procedure* While we recommend the utilization of salivary sampling measures, there is also a need to attentively control for influencing factors. Saliva samples are easily contaminated by factors such as gum health, food, or alcohol intake (Schultheiss & Stanton, 2009). Rigorous subject screening is advised to enhance the validity of findings. We propose a brief screening of potential sources of sample contamination upon collection, including information about caffeine consumption, use of medication, smoking, gum health, and last food consumption. Participants not meeting the criteria should be excluded. Upon sampling, subjects should be asked to rinse out their mouths with water and avoid drinking beverages for the remainder of the sampling procedure. We recommend a minimum of four saliva samples: (1) the baseline; (2) baseline + 20 min; (3) baseline + 40 min; and (4) baseline + 60 min. Immediately thereafter, the saliva samples should be frozen in a − 80 °C freezer visit, where they can be stored up to a year (Dabbs, 1991).
4. *Frequent and Timed Sampling* In order to capture the dynamic nature of hormonal fluctuations, future studies should carefully plan the timing and frequency of sample collections. While there is no consensus as to the most appropriate time of day for sampling, procedures in the early afternoon avoid morning peaks as well as steeper slopes in the first half of the day (Dickerson & Kemeny, 2004; Wirth et al., 2006). Additionally, samples should be collected at the same time from all subjects. Given the query of longitudinal synchronizations for this specific subject pool, future studies should aim for frequent and regular saliva collections. While the specific study designs should be determined by the respective researchers, we encourage weekly specimen collections on a minimum of at least two consecutive days for high significance.
5. *Quality Control Measures* Multiple specimen collection offers the unique opportunity to check for various influencing factors of the already impeded study of hormonal linkage. To ensure assay quality, each saliva sample should be assayed twice, and analyses should be flagged for sources of sample contamination, such as bleeding gums or lipstick. A wide variety of literature has proposed frameworks for fundamental assay quality parameters and measurement reliability (see Schultheiss & Stanton, 2009 for an example). We recommend the implementation of these frameworks.
6. *Analytical Strategy* To our knowledge, there is no standard analysis recommendation for the study of endocrinological adaptions and synchronizations. The use of multiple collections, however, can help account for and remove extreme outliers. Furthermore, we recommend including the time of each collection as a within-person covariate to account for diurnal fluctuations over the course of a day. While the choice of statistical analyses is up to the respective researchers, the hierarchical linear model (HLM) has frequently been utilized in hormonal research. HLM is well-suited for the analysis of repeated measurements over time, while allowing the exploration at both within-person (Level 1) and between-person levels (Level 2) (Hruschka et al., 2005). Additionally, HLM effectively models data in case of missing values and varying time intervals between measurement time points (Huta, 2014).

## Conclusion

All in all, the present review was able to synthesize a great number of records supporting the notion of synchronized parental endocrine systems both before and after childbirth. Expectant parents displayed linkages in cortisol, testosterone, and progesterone levels during the prenatal period. Further synchrony was reported for oxytocin, testosterone, and cortisol levels up to two years after birth. The functional significance of these covariations translates into the adaptation to parental behavior, the promotion of romantic bond, and intergenerational transmissions impacting infant development. The strong evidence of bi-directional effects of cortisol synchrony stresses the importance of viewing physiological synchrony as a complex process; the effects of which are often context-dependent. Future research should investigate postpartum covariations more thoroughly, especially with respect to their impact on postpartum health and infant development. In light of the limited and variable data in this field of study, we have, for future studies, proposed a framework that includes the utilization of standardized study protocols and repeated specimen collection. We believe that adherence to these standards would ensure the scientific rigor necessary to advance our understanding of the intricate dynamics of hormonal synchronizations. The identification of processes in which these synchronizations serve either an adaptive or a maladaptive purpose may provide a scientific basis for better postpartum health and adaptation.

## Data Availability

This review was based on published data. All data extracted for this study are included in the article.

## Appendix 1

See Table 3.

**Table 3:** Quality assessment of included studies, sorted alphabetically

References	CK1	CK2	CK3	CK4	CK5	CK6	CK7	CK8	CK9	CK10	CK11	CK12	CK13	CK14	Overall quality
Aviv et al. (2023)	Y	Y	Y	Y	Y	NA	N	NA	NA	NA	Y	NA	NA	Y	Fair
Berg & Wynne-Edwards (2002)	Y	Y	Y	Y	N	NA	NA	NA	NA	NA	N	NA	N	N	Poor
Braren et al. (2020)	Y	Y	Y	Y	Y	NA	N	NA	NA	NA	Y	NA	NA	Y	Good
Braren et al. (2021)	Y	Y	Y	Y	Y	NA	N	NA	NA	NA	Y	NA	NA	Y	Good
Cárdenas et al. (2023)	Y	Y	Y	Y	Y	NA	NA	NA	NA	NA	Y	NA	Y	Y	Good
Edelstein et al. (2015)	Y	Y	Y	Y	Y	NA	Y	NA	NA	NA	Y	NA	Y	Y	Good
Gordon et al. (2010)	Y	Y	Y	Y	Y	NA	Y	NA	NA	NA	Y	NA	Y	CD	Good
Gordon et al. (2017)	Y	Y	Y	Y	Y	NA	Y	NA	NA	NA	Y	NA	Y	Y	Good
Khaled et al. (2021)	Y	Y	Y	Y	Y	NA	Y	NA	NA	NA	Y	NA	Y	Y	Good
Saxbe et al. (2015)	Y	Y	Y	Y	Y	NA	Y	NA	NA	NA	Y	NA	Y	Y	Good
Saxbe et al. (2017)	Y	Y	Y	Y	Y	NA	Y	NA	NA	NA	Y	NA	Y	Y	Good
Sim et al. (2020)	Y	Y	Y	Y	Y	NA	Y	NA	NA	NA	Y	NA	Y	N	Good
Storey et al. (2000)	Y	Y	Y	Y	Y	NA	N	NA	NA	NA	Y	NA	NA	Y	Poor

Done according to quality assessment tools by National Institutes of Health (2019) for observational cohort and cross-sectional studies. Quality assessment tool answers. Y = yes, N = no, NA = not applicable, CD = cannot determine and NR = not reported

## Abbreviations

HLM: Hierarchical linear model
HPA axis: Hypothalamic–pituitary–adrenal axis
PPD: Postpartum depression
